# Topography-guided excimer laser ablation in refractive surgery

**DOI:** 10.3389/fopht.2024.1367258

**Published:** 2024-03-07

**Authors:** Şefik Can İpek, Canan Asli Utine

**Affiliations:** ^1^ Dunyagoz Suadiye Hospital, Istanbul, Türkiye; ^2^ Department of Opthalmology, Faculty of Medicine, Dokuz Eylül University, İzmir, Türkiye; ^3^ İzmir Biomedicine and Genome Center, İzmir, Türkiye; ^4^ Department of Ophthalmology, University of Naples Federico II, Napoli, Italy

**Keywords:** excimer, laser therapy, refractive surgery, keratoconus, ablation techniques, corneal topography

## Introduction

Over the last two decades, a notable evolution has occurred in customized laser ablations. The ablation profiles for conventional treatments do not consider the cornea’s asphericity and the eye’s higher-order aberrations. All conventional excimer laser profiles perform identical ablations in all patients with the same refractive error. Additionally, surgically induced corneal aberrations have been identified as contributing to decreased contrast sensitivity and issues such as glare, halos, and disturbances in night vision.

To achieve the best outcomes and enhance patient satisfaction, various “customized” ablation profiles have been introduced. These include wavefront-guided (WFG) and topography-guided (Topo-G) ablation, each offering a unique approach to addressing refractive errors. Topo-G ablation is distinct for its emphasis on topographical data to guide the laser reshaping of the cornea. The rationale behind Topo-G ablation is based on the cornea’s shape, and optical quality is intricately tied to the corneal topography. By taking into account the corneal surface’s unique characteristics, such as local irregularities and curvature variations, Topo-G ablation offers a tailored approach to corneal reshaping.

This review will explore the principles, clinical applications, outcomes, and comparative analyses associated with Topo-G excimer laser ablation. We will also delve into the importance of assessment and surgical planning clinical studies, technological advancements, and considerations that contribute to improving patient outcomes.

## Topography guided ablation in virgin corneas

### Principles

The cornea serves as the primary refractive surface of the eye and is responsible for a significant amount of ocular aberrations. Conventional excimer laser ablation profiles perform identical ablations in all patients with the same refractive error, resulting in corneal HOA induction (1, 2). While wavefront-optimized ablation profiles aim to reduce treatment-induced spherical aberrations, they do not correct the underlying higher-order aberrations. Topo-G treatments aim to achieve optimal corneal curvature based on corneal elevation data (3). The basic principle of this treatment is to achieve optimal corneal curvature with laser ablation to flatten the steep areas of the cornea and steepen the flat areas. Using both myopic and hyperopic ablation patterns for similar correction reduces the need for stromal ablation (4).

The primary objective of Topo-G treatment in virgin corneas is to regularize the corneal surface, reduce higher-order aberrations (HOAs) resulting from excimer ablation, alleviate night vision issues, decrease subjective complaints from patients due to irregular optics, and typically improve postoperative uncorrected distance visual acuity (UDVA) compared to preoperative corrected distance visual acuity (CDVA).

Topographical irregularity is observed in 10-40% of corneas that are considered normal and have not been diagnosed with ectatic disease. Irregular patterns such as asymmetrical bow ties, inferior and superior steeping, skewed radial axis, and asymmetric bow ties can be observed in such corneas (5–9). The primary higher-order aberrations (HOAs) present are coma and trefoil. Conventional excimer ablations may cause deterioration in postoperative visual quality, night vision problems, and an increase in preoperative irregularities, especially in asymmetric corneas.

### Clinical approach

Clinical refraction is a standard correction method in most laser vision correction treatments. However, there may be instances where refractive data differs from topographic data, particularly regarding the magnitude and axis of astigmatism (3). The difference between manifest refractive astigmatism and anterior corneal topographic astigmatism is defined as ocular residual astigmatism (ORA). Possible factors causing ORA include anterior corneal HOAs, posterior corneal astigmatism and posterior corneal HOAs, lenticular astigmatism, lens decentration and tilt, retinal astigmatism, and cortical perception. However, in a study of 37,454 eyes, no correlation was found between coma aberration and ORA in virgin corneas (10). Contrary to popular belief, the power and axis of manifest refractive astigmatism may not correlate with coma, especially in virgin corneas. Also, ORA magnitude varies according to the orientation of the astigmatism. In most eyes with WTR astigmatism, refractive astigmatism is lower than corneal astigmatism; whereas, in most eyes with ATR astigmatism, refractive astigmatism is greater than corneal astigmatism. This relationship is related to posterior corneal astigmatism (11). Indeed in Topo-G treatments, whether to treat manifest clinical astigmatism or topographically based anterior corneal astigmatism is controversial. This is particularly significant when dealing with high astigmatic axis and dioptric discrepancies.

Several planning algorithms have been developed for the Alcon Contoura Topo-G treatment system. Firstly, the FDA algorithm uses manifest refraction, while a modified FDA algorithm determines the astigmatic axis of the correction based on corneal topographic data. However, the FDA algorithm needs to satisfy the following conditions:

1. For patients with astigmatisms of ≥ 2.00 D, the difference in the astigmatic axis between manifest refraction and topographic data should not exceed 5 degrees.
2. For patients with astigmatisms of ≤ 1.75 D, the difference in the astigmatic axis between the manifest refraction and topographic data should not exceed 10 degrees.
3. The difference in astigmatic power between manifest refraction and topographic data should not exceed 0.75 D.

According to Alcon guidelines, if the above criteria are not met, WFO ablation is recommended. If the subjective astigmatism power is greater than the computed astigmatism power, it is recommended that the calculated astigmatism power and axis are used and that the spherical equivalent is equal to the subjective spherical equivalent. If the subjective astigmatism power is less than the computed astigmatism power, it is recommended to enter a value between the subjective and calculated power to use the computed axis and that the spherical equivalent is equal to the subjective spherical equivalent.

The FDA study on primary Topo-G treatment involved 249 patients. 32% of eyes achieved a UDVA of 20/12.5 or better, 69% achieved a UDVA of 20/16 or better, and 93% achieved a UDVA of 20/20 or better. In 30% of eyes, postoperative UDVA was higher than preoperative CDVA (12). The reason for such high clinical and refractory success may be the strict inclusion criteria of the FDA study. Only symmetric normal corneas were included in the study, and patients with atypical corneal topography were excluded. Only eyes with low ORA were included in the FDA study.

Another planning algorithm has been developed by Kanellopoulos et al. The topography-modified refraction (TMR) technique combines the corneal astigmatic power and axis obtained from topography with the manifest refraction to plan laser treatment. When different from the clinical refraction, topographic adjustment of the amount and axis of astigmatism treated may offer superior outcomes in topography-guided myopic LASIK. The corneal topography software calculates the topographic ablation pattern, which provides the astigmatism and axis to be corrected with the ablation. It also calculates the potential amount of spherical change resulting from this correction. Kanellopoulos et al. developed a method for correcting refractive astigmatism that differs from topographic astigmatism. The correction is made based on the power and axis of the topographic astigmatism. Any spherical changes that occur are added to the spherical ablation. In a contralateral eye study of 100 eyes from 50 patients with Topo-G LASIK ablation, residual refractive astigmatism over 0.5 D at three months was 11.7% in the TMR group and 27.8% in the manifest refraction group. The difference was statistically significant (13).

A retrospective analysis of 1274 eyes revealed comparable results for both methods in astigmatic axis discrepancies below 20°. Nevertheless, topographically based astigmatic correction caused inferior refractive and visual outcomes and demanded more retreatment when axis discrepancies exceeded 20° (14). A more extensive study analyzed retrospective data from 25,396 eyes that underwent Topo-G laser based on manifest refraction. A large preoperative discrepancy (45° to 90°) between refractive astigmatism and topography-measured anterior corneal astigmatism does not negatively impact topo-G LASIK. Eyes with a large discrepancy (45° to 90°) have identical refractive and visual outcomes compared to eyes with a slight discrepancy. The authors suggest that the astigmatic axis should be based on manifest refraction when planning treatment for these eyes (15). In a prospective study involving 64 eyes, it was reported that using the manifest refractive astigmatic axis in topo-guided ablation treatment for astigmatism resulted in more successful visual and refractive outcomes than the topography-based anterior corneal astigmatic axis (16).

The Layer Yolked Reduction of Astigmatism Protocol (LYRA) protocol is another planning algorithm for Topo-G laser ablation. According to this protocol, Contoura measured astigmatism power and axis more accurately and corrected astigmatic power and axis to create a more uniform, aberration-free cornea. They theorized that the HOAs of the anterior cornea contribute to changing the astigmatic axis and power that a patient will subjectively accept to achieve an acceptable quality of vision. The author has theorized that there is a link (or yolk) between HOA removal and astigmatism correction and that the Contoura processing software can accurately analyze this linkage and using the manifest refraction with Contoura leaves out this critical link; that is, the removal of HOAs affects the astigmatism of the refractive correction layer. In their study of 50 eyes, 80.85% of patients had vision 20/15 and better, and 100% were 20/20 or better. They stated that although there was a significant deviation between the measured and manifest astigmatic power (over 0.5 D) and axis (14.94°), astigmatism was completely treated in 48 out of 50 eyes (17). in a comparative contralateral eye study of 64 eyes of 32 patients in turkey, Topo-G ablation using the LYRA protocol was compared with WFO ablation. Visual results showed similar success rates in both groups. However, the Topo-G group had less vertical and horizontal coma induction and less tissue ablation than the WFO group (18).

The Phorcides Analytic Engine is an analysis and planning program that considers various factors when determining the optimal treatment for an eye with topography-guided LASIK. These factors include anterior corneal astigmatism, topographic irregularities that create higher-order aberrations, posterior corneal astigmatism, and lenticular astigmatism. Lobanoff et al. compared the results of the group treated according to manifest refraction with those treated according to Phorcides analysis. Residual refractive results, both sphere and cylinder, were similar between groups. However, significantly more eyes had 20/16 or better-uncorrected distance visual acuity (62.5% Phorcides, 41.3% manifest) (19). A study of 52 eyes compared manifest refraction, topographic refraction, and Phorcides analysis. The study reported that the Phorcides group had the most successful results in terms of residual refractive error (20). In a recent prospective study on Phorcides, 135 eyes of 65 patients treated with Topo-G using the analysis program were examined. At three months postoperatively, 100%, 89%, and 28% of eyes achieved UDVA of 20/20, 20/15, and 20/12.5 or better, respectively. 92% of eyes had postoperative UDVA equal to or better than their preoperative CDVA (21).

Several alternative planning algorithms have been developed for Topo-G treatments, but each has limitations (22, 23). As surgeons, staying current is essential to achieving the best refractive results. The success of refractive outcomes has increased with Topo-G treatments, which have become increasingly popular in recent years. These treatments allow us to correct corneal HOAs and are more effective in treating high astigmatism. Large-scale comparative prospective studies are needed to determine which method will help us achieve the most successful refractive results.

## Advantages and disadvantages

The main advantages and disadvantages of topo-guided treatments compared to wavefront-guided treatments can be listed as follows:

1. Corneal curvature measurements are independent of pupil diameter, unlike wavefront measurements. Therefore, topography-guided treatments are independent of pupil centroid shift errors and accommodative status of the eye (3, 4, 24).
2. In topography-based treatments, centralization is at the corneal apex and independent of angle kappa errors (3, 24).
3. Compared to wavefront measurements, many points on the cornea are measured. This better assesses the peripheral cornea, which is responsible for most HOAs (3, 12).
4. It allows treatment of corneas where wavefront ablation is inaccurate due to corneal opacity or irregularity. This makes the topographically acquired data a far more stable parameter than the wavefront data (12, 24).
5. As only corneal HOAs are corrected, the appearance of possible internal (lenticular) HOAs, previously masked by corneal HOAs, may lead to reduced visual quality (3).
6. Corneal topographers do not provide information about the spherocylindrical refraction of the eye. Therefore, more than topographic data is needed for treatment planning. Manifest refraction should also be used in combination with this data (12, 24).

## Comparison with other ablation profiles

Studies comparing Topo-G with other ablation profiles for myopia and astigmatism have demonstrated comparable or superior clinical outcomes (**Table 1**).

**Table 1:** Prospective comparative studies comparing Topo-G ablation with WFO ablation.

Author, Year	Number of eyes	Follow-up(months)	Preop SE (D)	Preop Astigmatism (D)	Postop SE (D)	Postop Astigmatism (D)	Comments
El Awady,2011 (25)	42 WFO	6	-5,42 ± 3.7	-1.80 ± 1.15	-0,2 ± 0.07	-0,64 ± 0,63	*Topo-G group had statistically significantly higher UCVA and postoperative HOA of the Topo-G group was smaller than that of the WFO group
	42 Topo-G		-5,23 ± 1.6	-1,94 ± 1.40	-0,016 ± 0,057	-0,5 ± 0,23	
Shetty, 2017 (26)	30 WFO	6	-5,08 ± 2.5	-1.10 ± 0.76	-0,50 ± 0,53	-0,53 ± 0,27	*Topo-G group had less corneal asphericity change and lower aberrations.
	30 Topo-G		-4,93 ± 2.47	-1.08 ± 0.71	-0,41 ± 0,49	-0,51 ± 0,23	
Jain, 2016 (4)	35 WFO	6	-3,89 ± 1.85	-0,29 ± 0,42	-0,17 ± 0,38	No data	* Topo-G ablation group had better contrast sensitivity, less HOA induction, and less stromal ablation.
	35 Topo-G		-4,19 ± 1.92	-0,30 ± 0,38	-0,02 ± 0,29	No data	

In a prospective randomized controlled contralateral eye study of 84 eyes, the Topo-G and WFO ablation profiles for myopia were compared. The six-month postoperative controls indicated that the Topo-G treatment group had statistically significantly higher UCVA. The postoperative spherical equivalent of the WFO group was -0,2 ± 0,07 D, whereas that of the Topo-G group was -0,016 ± 0,057 D. Additionally, the postoperative HOA of the Topo-G group was smaller than that of the WFO group, but the difference was not statistically significant. There was a decrease in most of the individual terms of HOAs in the Topo-G group, but it was only statistically significant in a vertical coma. The authors speculated that both ablation profiles provided good refractive results, but the Topo-G ablation induced fewer HOAs (25).

A prospective contralateral eye study from India compared Topo-G and WFO ablation in 60 eyes of 30 patients. Postoperative controls at six months showed similar visual gains in both groups, with less corneal asphericity change and lower aberrations in the Topo-G group (26).

Jain et al. compared Topo-G and WFO treatments in a 35-patient contralateral eye study and found that the Topo-G ablation group had better contrast sensitivity, less HOA induction, and less stromal ablation (4).

A recent meta-analysis compared Topo-G and WFO LASIK treatments to treat myopia with and without astigmatism. A total of 1168 eyes participating in 7 randomized controlled trials were included in the study. There were no statistically significant differences in the uncorrected distance visual acuity ratio. Topo-G LASIK exhibited more accurate postoperative refraction predictability and less surgically induced higher-order aberrations, spherical aberrations, and coma (27).

In another recent meta-analysis, the study compared Topo-G and wavefront-optimized treatment profiles. The analysis included a total of 1,425 eyes from 11 studies. No statistically significant differences were observed in the proportion of eyes achieving uncorrected distance visual acuity between Topo-G and WFO ablation procedures. After Topo-G ablation, a significantly higher proportion of patients’ eyes achieved postoperative refraction within ±0.5 diopter of the target refraction compared to those undergoing WFO ablation. The higher-order aberrations, spherical aberration, and coma were significantly lower in the Topo-G group (28).

Studies comparing SMILE and Topo-G treatments are also available in the literature. In a prospective randomized contralateral eye study by Kanellopoulos et al. in 2017, SMILE and Topo-G treatments were compared to treat myopia and myopic astigmatism. 44 eyes of 22 patients were included in the study. In the methodology of the study, the astigmatic axis and power in the Topo-G treatment group were modified according to the topographically measured astigmatic axis and power. Three months postoperatively, 86,4% of the Topo-G group and 68,2% of the SMILE group had UDVA of 20/20, and 59,1% and 31,8%, respectively, had UDVA of 20/16. The difference is statistically significant in favor of the Topo-G. Also, the preoperative CDVA vs postoperative UDVA difference indicated that 9,1% of the eyes in the Topo-G group and 4,5% of the eyes in the SMILE group gained two lines. The Topo-G group was statistically significantly more successful when both methods were compared regarding residual manifest spherical equivalent and residual refractive astigmatism. The authors speculated that Topo-G LASIK was superior to SMILE in all visual performance parameters studied, both subjective and objective (29).

In a study conducted by Yang et al. on 46 eyes, the clinical efficacy of Topo-G LASIK and SMILE treatment was compared. The study findings demonstrated that both treatments had similar clinical outcomes after three months. However, the Topo-G LASIK group exhibited better early postoperative UCVA and better contrast sensitivity in scotopic conditions (30).

## Topography guided ablation for ectatic and irregular corneas

Topography-based ablation aims to regularize corneal irregularities. The literature provides various cases, including ectatic corneal diseases, iatrogenic ectasias, minor or decentered optic zone treatment, post-keratoplasty cases, and diseases causing corneal irregularity.

## Post-refractive surgery complications

Decentralized ablation is a rare complication of refractive laser surgery. Clinical complaints include visual distortion, astigmatism, halo, glare, monocular diplopia, and loss of visual acuity. The diagnosis is made by identifying the decentralized pattern of topography. Low decentration can negatively impact contrast visual acuity and lead to higher-order aberrations. On the other hand, decentration of more than 1.0 mm can significantly compromise visual performance (31). Nowadays, the incidence has decreased with the development of eye tracker and laser systems. In a case series of 11 eyes, Kymionis et al. reported that treatment of decentralized ablation with Topo-G ablation improved uncorrected and corrected visual acuity (32). In a retrospective case series, it was shown that topographically based treatment was effective and safe in the treatment of decentralization following refractive surgery complication (33).

A slight optic zone can result in complaints such as decreased visual function, halo, glare, and monocular diplopia, particularly in mesopic conditions. In the first generation of laser treatments, the optic zone was kept small to reduce stromal ablation, especially in high myopic treatments. If such complaints develop, reshaping the optic zone with a Topo-G laser to enlarge it can be helpful. Enlarging the optical zone guided by topography has been demonstrated to be more effective in improving corneal surface regularity and reducing patient symptoms (33, 34).

The combination of Topo-G laser and CXL can also be used to treat iatrogenic ectasias. Studies have shown that this treatment is effective and safe in halting the progression of ectasia and providing refractive and topographic stability, particularly in cases where it occurs after refractive surgeries such as LASIK (33, 35).

The Topo-G laser can also be used in cases of decentralization due to SMILE. An increase in visual acuity and a decrease in HOA were observed with Topo-G retreatment in a patient with visual complaints due to decentralization after SMILE surgery (36).

## Keratoconus and ectatic corneal disorders

Corneal crosslinking therapy is a significant breakthrough in the treatment of keratoconus patients. This therapy has significantly reduced the need for corneal transplantation in such patients (37, 38). The increased strength of the collagen fibers in the cornea prevents disease progression and vision loss (39). The effectiveness of cross-linking treatment has led to the hypothesis that corneal irregularities can also be regularized in combination with excimer laser ablation. Research has demonstrated that irregular astigmatism can be reduced and best-corrected visual acuity (BCVA) can be improved with CXL + Topo-G PRK ablation (40–42).

Kanellopoulos was the first to report sequential CXL and final Topo-G ablation for the treatment of keratoconus in 2007 (43). After the success in this case and the demonstration of stability in a follow-up period of up to 30 months, successful results have been reported by the same author and others from all over the world, and different techniques have been described (44–47).

Although many studies describe different techniques, there still needs to be generally accepted methods. There is still controversy about whether treatment should be performed sequentially or simultaneously. Both approaches have advantages and disadvantages. In recent years, the simultaneous approach has been used more frequently. The advantages and disadvantages of simultaneous treatment can be listed as follows (3, 48, 49):

1. Less time off work and treatment in a single session
2. Keratocyte apoptosis caused by cross-linking is thought to reduce haze formation.
3. Removal of Bowman’s membrane during ablation increases riboflavin penetration into the stroma
4. The tissue ablation rate can be accurately estimated Because the virgin cornea is ablated during the simultaneous treatment.
5. Sequential treatments ablate previously cross-linked cornea, so biomechanical resistance may be less.

The disadvantages of simultaneous treatment are that corneal flattening and refractive changes may occur after CXL. In addition, CXL may cause excessive haze formation in some patients. Rarely there are patients who show progression despite CXL. Sequential treatment may be considered in these patients.

## Patient selection

CXL treatment is recommended for all patients diagnosed with progressive keratoconus, regardless of age. In addition, it is reported that patients under the age of 35 may undergo CXL if the risk of progression is assessed at the time of initial diagnosis. The minimum corneal thickness recommended for patients undergoing combined CXL and Topo-G PRK is 450 microns (33, 46, 48, 49). The maximum ablation depth is recommended to be 50 microns. This value is arbitrary and based on the clinical experience of the authors (48). The minimum intraoperative stromal bed after maximum correction is recommended to be 350 microns. Ideal candidates for TG-PRK + CXL have a dioptric difference of less than 10 D across their cornea, calculated within a 6mm optical zone by measuring the difference between the flattest and steepest areas (47).

The localization of the cone is also effective in the success of treatment. One study compared two groups of patients who underwent Topo-G PRK and CXL for cone location and found that visual success was superior in the group in which the cone was located in the central 2 mm zone (50).

The primary objective is to achieve corneal regularization rather than refractive correction. Therefore, setting the optic zone at 5.5 mm is recommended to minimize tissue ablation. The aim is to correct a maximum of 70% of the spherical and astigmatic errors found. It is recommended that this correction is at most 50 microns (33, 44, 48).

This treatment is commonly used for younger patients with progressive keratoconus. It may also be used for stable keratoconus patients who cannot tolerate contact lenses. Older keratoconus patients who require cataract surgery may be preferred for corneal regularization before surgery. The procedure is not recommended for patients with corneal scarring, a history of herpes, or a high dioptric difference of 10D in the cornea due to excessive ablation. Additionally, it is not suitable for very thin corneas or advanced diseases (47).

**Figure 1:**
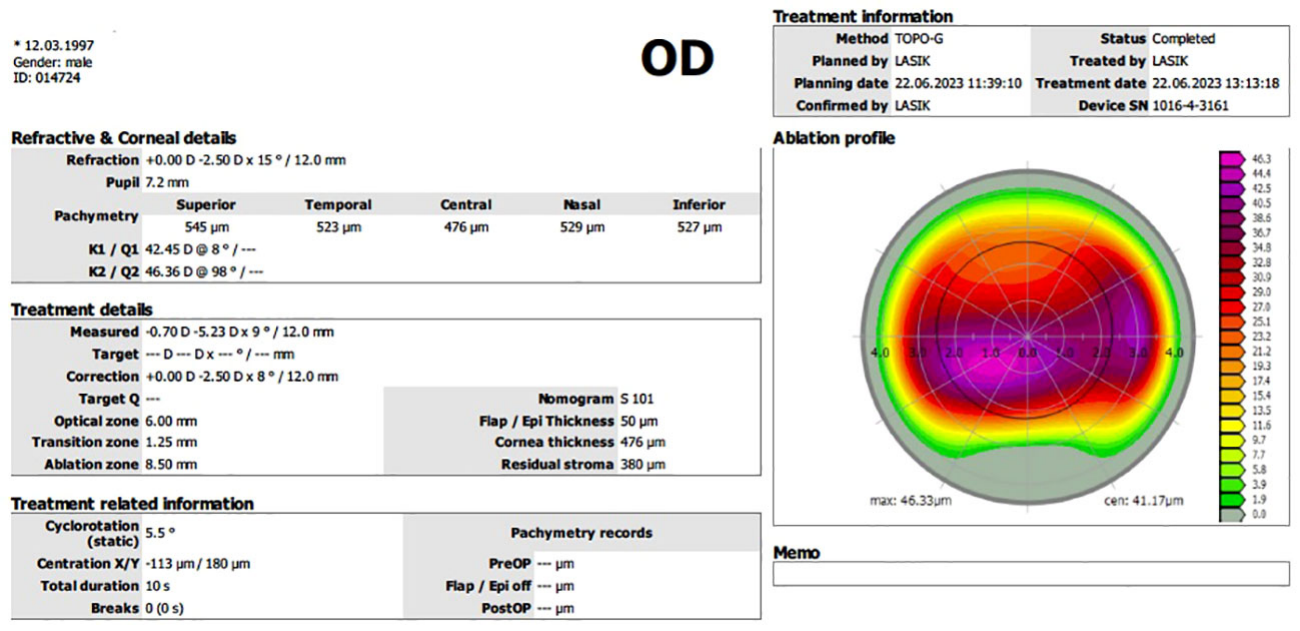
The final treatment plan included a 46-micron ablation for corneal regularisation with partial cylindrical correction. The plan was to flatten with myopic ablation over the cone and steepen with hyperopic ablation in the mid periphery.

It is essential to obtain sufficient high-quality topographical scans before treatment. Once at least six similar scans have been taken, treatment planning begins. The first step is to consider the ablation profile to be used to correct higher-order aberrations without considering refractive correction. To minimize stromal ablation, refractive correction is planned as an under-correction (**Figure 1**).

**Figure 2:**
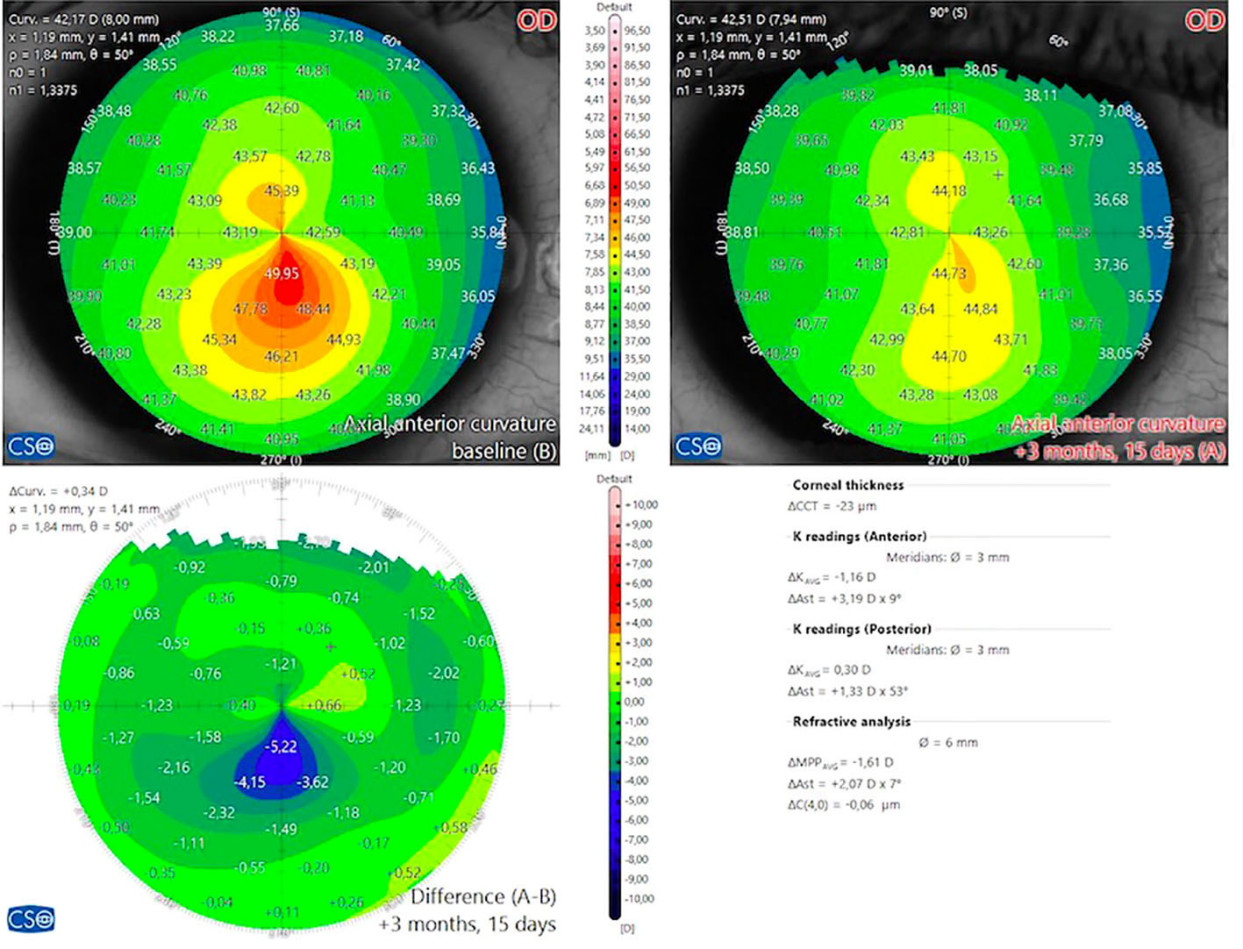
Day 45 patient appearance with asymmetric bow tie-like topography on the preoperative axial map after combined topo-G and CXL treatment. The effect of ablation can be seen in the difference map.

## Surgical technique and parameters

In the original Athens protocol, after insertion of the lid speculum, the epithelium is exposed to 20% alcohol for 20 seconds and peeled with a sponge. After recognition by the eye tracker and cyclotorsion compensation, excimer laser ablation is performed aligned to the corneal apex. After 0.02% mitomycin solution was applied to the ablated tissue for 20 seconds, it was washed with 10 ml cold BSS solution; then, the stroma was saturated with 0.1% riboflavin solution every 2 minutes for 10 minutes. CXL is then applied using a UVA beam with a wavelength of 370 nm and an irradiance of 3 mW/cm (2) at a distance of 2.5 cm for 30 minutes (48). Day 45 patient appearence with keratoconus after combined topo-G and CXL treatment (**Figure 2**).

## Epithelium removal

Several studies showed that, in the keratoconic cornea, the epithelium is thicker over the depressed stroma and thinner over the cone, so topography-guided ablation, applied to the stroma, can correct only the irregularity that the epithelium has not compensated (51, 52). Manual epithelial removal allows debridement of variable epithelial thickness with resultant underlying contours that vary from preoperative epithelium on measurements.

For these reasons, some authors have suggested using PTK mode during the epithelial debridement stage to eliminate the compensatory effect of the epithelium. This ensures a uniform tissue ablation in the selected optical zone, resulting in underlying tissue similar to the preoperative topography (49, 53).

## Long term results

In 2019, the 10-year results of 144 eyes treated with the Athens protocol were published. These results show that patients’ refractive and topographic improvement was maintained over ten years. Ectasia stabilization was achieved in 94.4% of patients. Progressive hyperopic shift was observed in 3.5% of patients (54).

A review published in 2020 analyzed the literature on combined excimer laser and CXL treatment for keratoconus, with a total of 479 eyes included in the review. The literature suggests that CDVA, UDVA, and HOA in mild to moderate keratoconus patients improved with combined treatment without compromising the biomechanical stability of the cornea, but longer follow-up studies are needed (42).

## Author contributions

SI: Writing – original draft, Writing – review & editing. CU: Writing – original draft, Writing – review & editing.
